# Supplementary figures and images for: Gender, caste, and heterogeneous farmer preferences for wheat varietal traits in rural India
Source: PLoS One. 2022 Aug 11;17(8):e0272126. doi: 10.1371/journal.pone.0272126 (PMC9371340; doi:10.1371/journal.pone.0272126)

**Appendix 1. Example Choice Card**


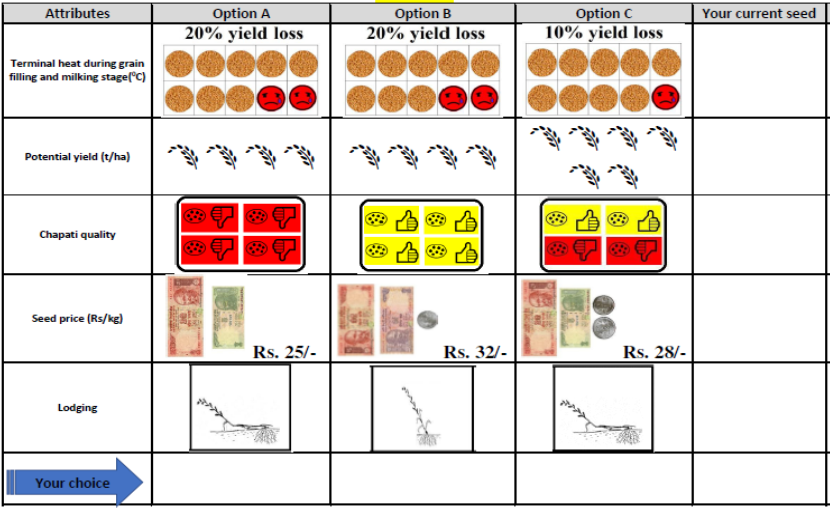

Supplement: S1 Appendix — (DOCX) [file pone.0272126.s001.docx]
