# Supplementary material for: Gender, caste, and heterogeneous farmer preferences for wheat varietal traits in rural India
Source: PLoS One. 2022 Aug 11;17(8):e0272126. doi: 10.1371/journal.pone.0272126 (PMC9371340; doi:10.1371/journal.pone.0272126)
Supplement: S2 Appendix — (DOCX) [file pone.0272126.s002.docx]

**Appendix 2. The Extent of Involvement of Male and Female Members in Wheat Varietal Selection**

|  | Share of households in which the decision on varietal selection is taken by | | | Had any discussion on varietal selection occurred among the household members? [1 = yes; 0 = no] | If discussion occurred, did it include women members of the household?  [1 = yes; 0 = no] |
| --- | --- | --- | --- | --- | --- |
|  | Male members | Female members | Jointly |  |  |
| *According to male respondents* |  |  |  |  |  |
| Overall (n = 418) | 0.971 | 0.017 | 0.012 | 0.880 | 0.321 |
| SCST (n = 130) | 0.969 | 0.015 | 0.015 | 0.854 | 0.369 |
| Other castes (n = 288) | 0.972 | 0.017 | 0.010 | 0.892 | 0.300 |
| *According to female respondents* |  |  |  |  |  |
| Overall (n = 414) | 0.935^**^ | 0.036^*^ | 0.027 | 0.836^*^ | 0.456^***^ |
| SCST (n = 133) | 0.917^*^ | 0.053^*^ | 0.030 | 0.727^***^ | 0.462 |
| Other castes (n = 281) | 0.946 | 0.029 | 0.025 | 0.890 | 0.453^***^ |

*Notes*: Estimates are obtained from the 2018 (first-round) farm-household survey conducted among the sample households of the present study. Figures in the parentheses (n) show the number of observations. ^**^ and ^***^ denote statistical significance of difference from the male group at the 0.05 and 0.01 levels, respectively.
