# Supplementary material for: Gender, caste, and heterogeneous farmer preferences for wheat varietal traits in rural India
Source: PLoS One. 2022 Aug 11;17(8):e0272126. doi: 10.1371/journal.pone.0272126 (PMC9371340; doi:10.1371/journal.pone.0272126)
Supplement: S1 File — (PDF) [file pone.0272126.s005.pdf]

## Card No. 1

| Attributes | Option A | Option B | Option C | Your current seed |
|---|---|---|---|---|
| Terminal heat during grain filling and milking stage($^0$C) | 10% yield loss 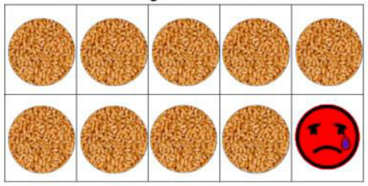 | 10% yield loss 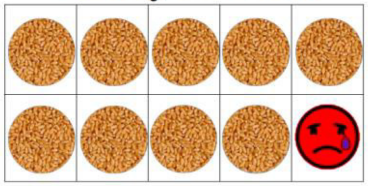 | 20% yield loss 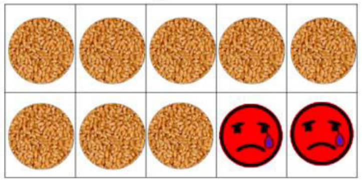 | |
| Potential yield (t/ha) | 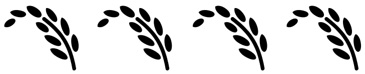 | 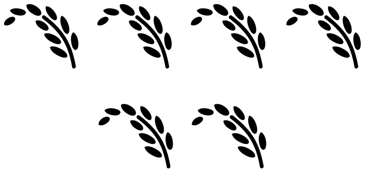 | 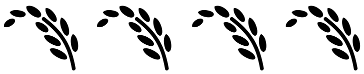 | |
| Chapati quality | 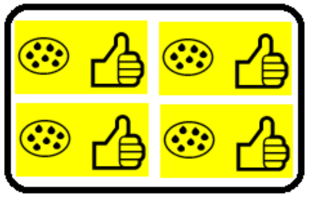 | 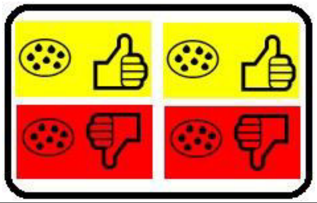 | 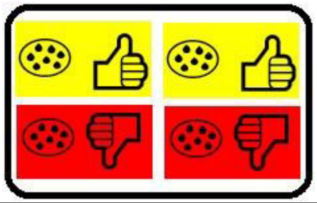 | |
| Seed price (Rs/kg) | 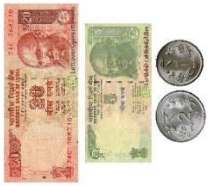 Rs. 28/- | 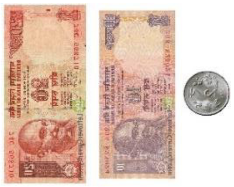 Rs. 32/- | 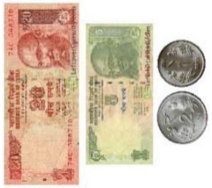 Rs. 28/- | |
| Lodging | 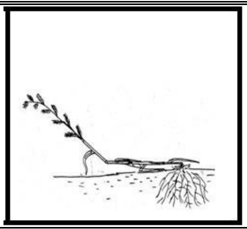 | 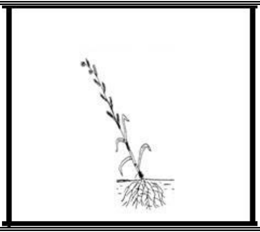 | 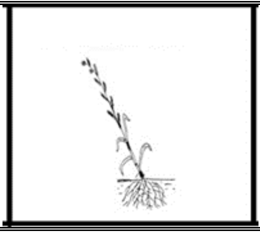 | |
| Your choice | | | | |

## Card No. 2

| Attributes | Option A | Option B | Option C | Your current seed |
|---|---|---|---|---|
| Terminal heat during grain filling and milking stage($^0$C) | 20% yield loss 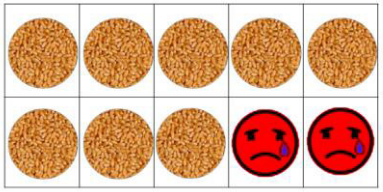 | 20% yield loss 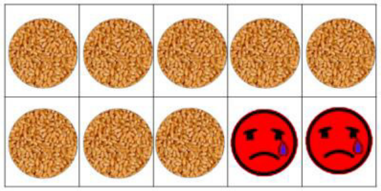 | 10% yield loss 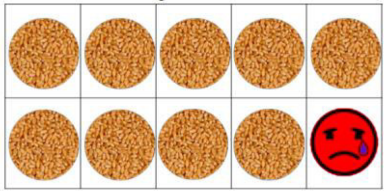 | |
| Potential yield (t/ha) | 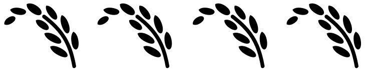 | 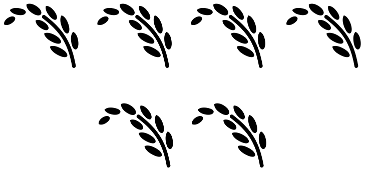 | 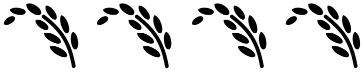 | |
| Chapati quality | 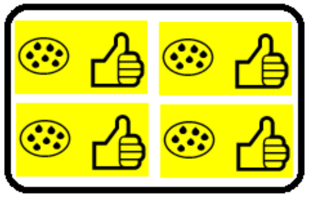 | 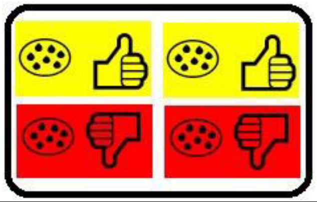 | 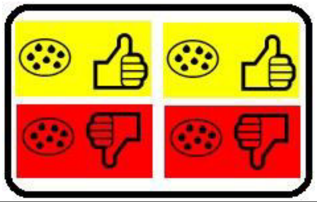 | |
| Seed price (Rs/kg) | 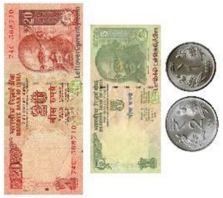 Rs. 28/- | 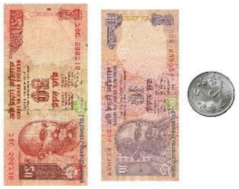 Rs. 32/- | 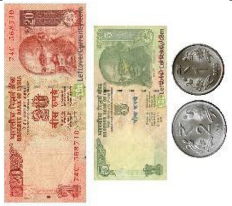 Rs. 28/- | |
| Lodging | 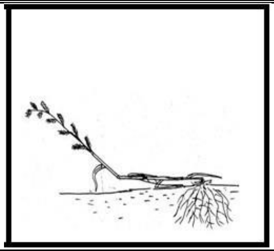 | 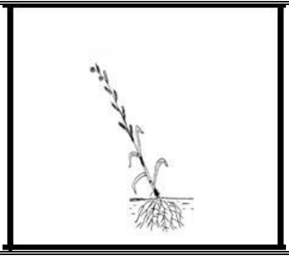 | 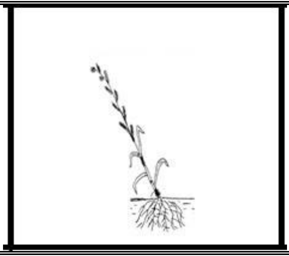 | |
| Your choice | | | | |

## Card No. 3

| Attributes | Option A | Option B | Option C | Your current seed |
|---|---|---|---|---|
| Terminal heat during grain filling and milking stage($^0$C) | 20% yield loss 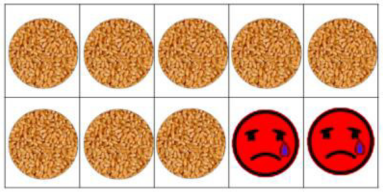 | 20% yield loss 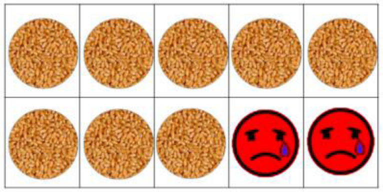 | 10% yield loss 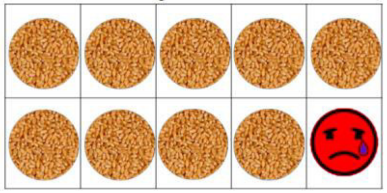 | |
| Potential yield (t/ha) | 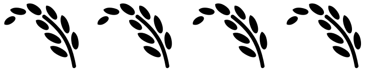 | 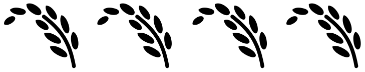 | 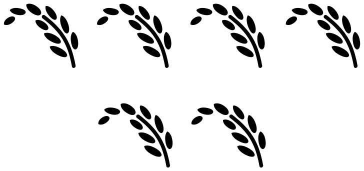 | |
| Chapati quality | 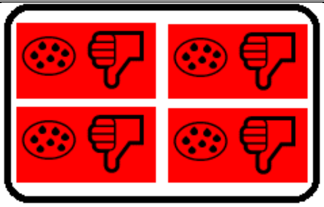 | 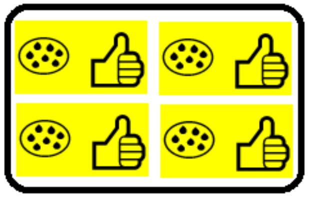 | 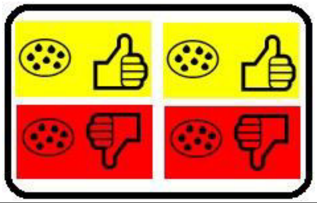 | |
| Seed price (Rs/kg) | 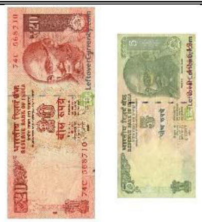 Rs. 25/- | 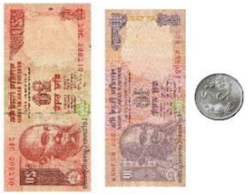 Rs. 32/- | 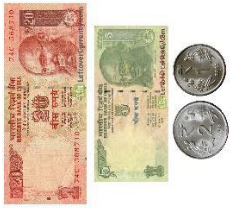 Rs. 28/- | |
| Lodging | 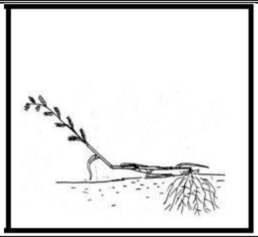 | 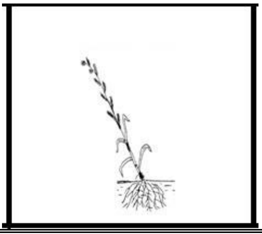 | 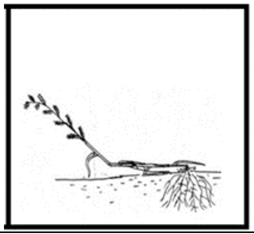 | |
| Your choice | | | | |

# Card No. 4

| Attributes | Option A | Option B | Option C | Your current seed |
|---|---|---|---|---|
| Terminal heat during grain filling and milking stage($^0$C) | 10% yield loss 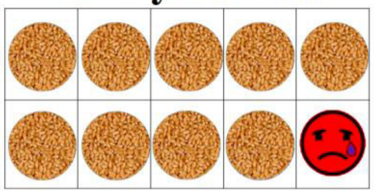 | 30% yield loss 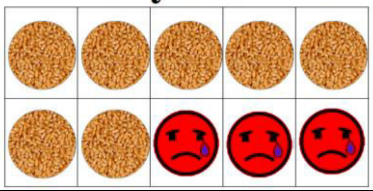 | 20% yield loss 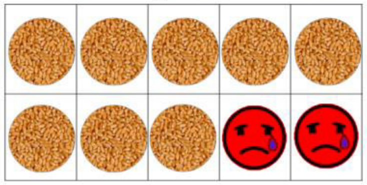 | |
| Potential yield (t/ha) | 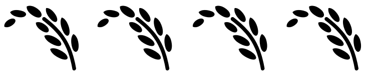 | 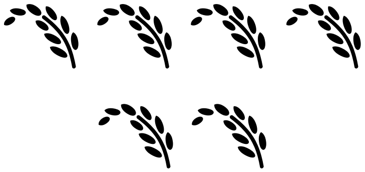 | 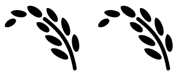 | |
| Chapati quality | 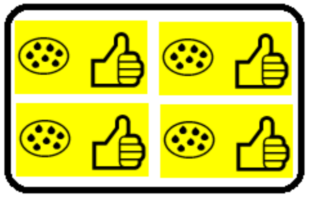 | 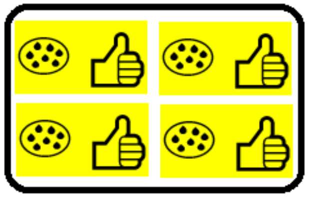 | 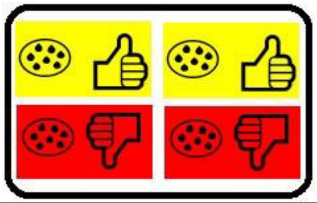 | |
| Seed price (Rs/kg) | 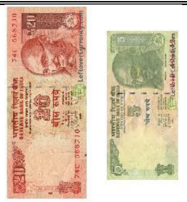 Rs. 25/- | 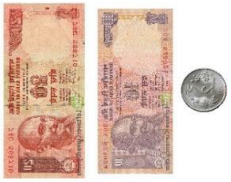 Rs. 32/- | 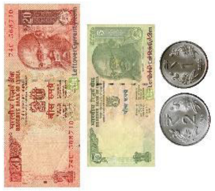 Rs. 28/- | |
| Lodging | 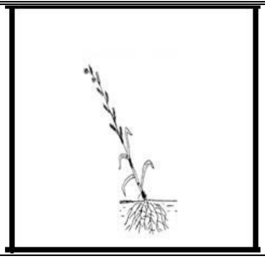 | 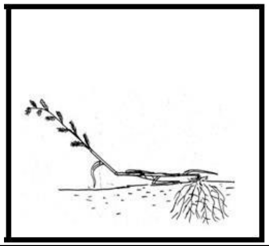 | 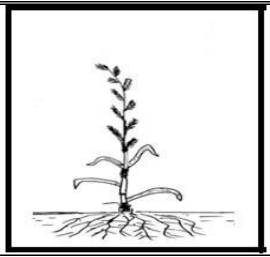 | |
| Your choice | | | | |

Card No. 5

| Attributes | Option A | Option B | Option C | Your current seed |
|---|---|---|---|---|
| Terminal heat during grain filling and milking stage($^0$C) | 20% yield loss<br>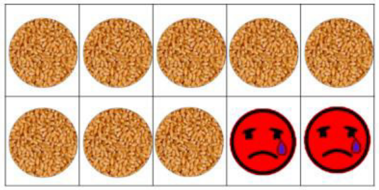 | 30% yield loss<br>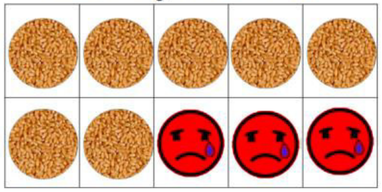 | 10% yield loss<br>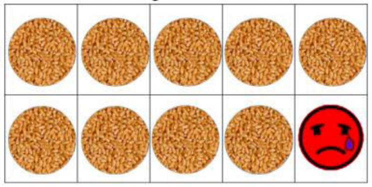 | |
| Potential yield (t/ha) | 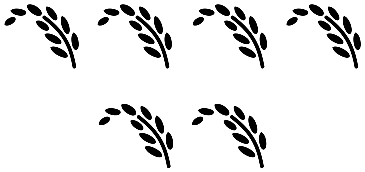 | 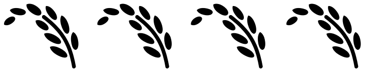 | 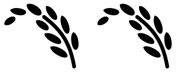 | |
| Chapati quality | 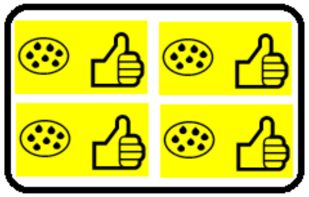 | 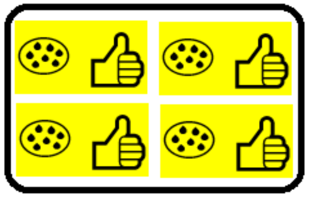 | 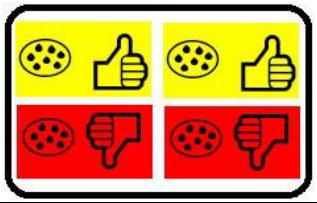 | |
| Seed price (Rs/kg) | 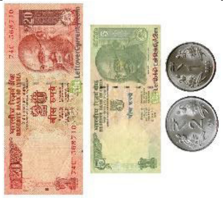 Rs. 28/- | 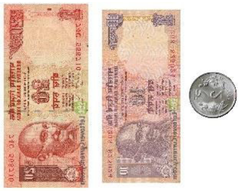 Rs. 32/- | 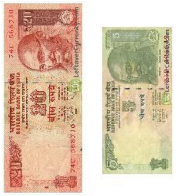 Rs. 25/- | |
| Lodging | 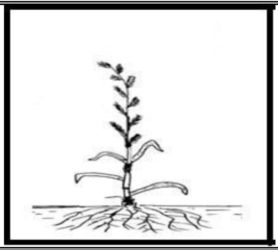 | 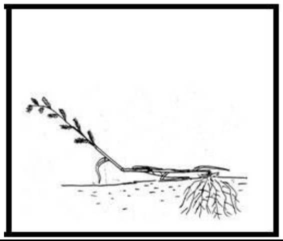 | 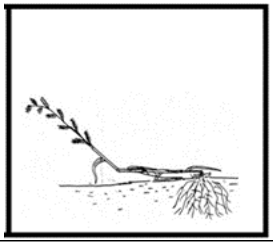 | |
| Your choice | | | | |

## Card No. 6

| Attributes | Option A | Option B | Option C | Your current seed |
|---|---|---|---|---|
| Terminal heat during grain filling and milking stage($^{0}$C) | 10% yield loss 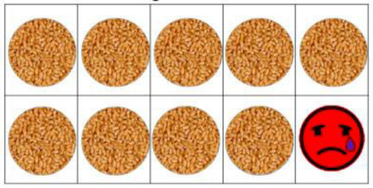 | 20% yield loss 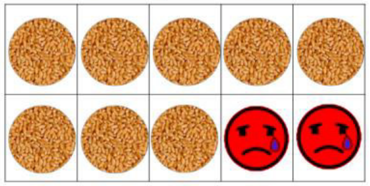 | 30% yield loss 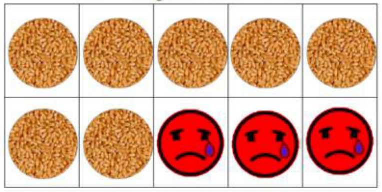 | |
| Potential yield (t/ha) | 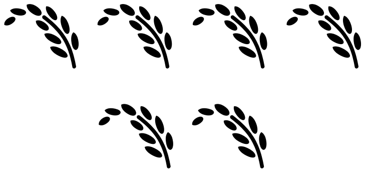 | 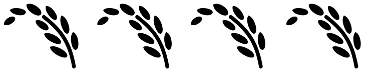 | 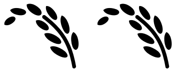 | |
| Chapati quality | 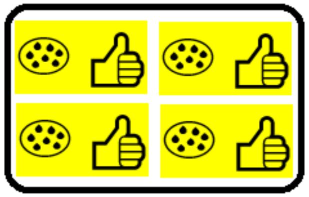 | 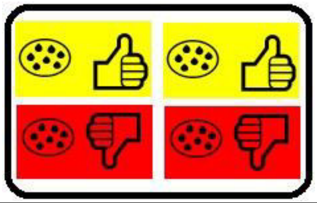 | 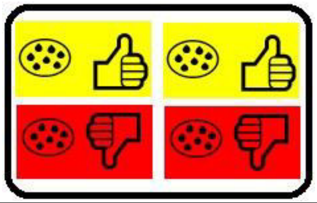 | |
| Seed price (Rs/kg) | 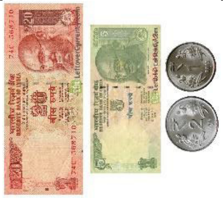 Rs. 28/- | 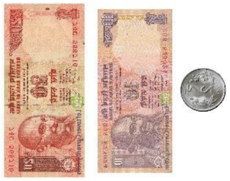 Rs. 32/- | 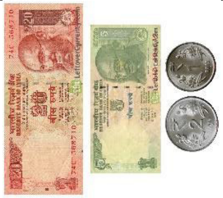 Rs. 28/- | |
| Lodging | 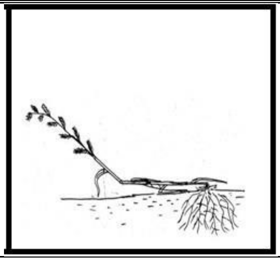 | 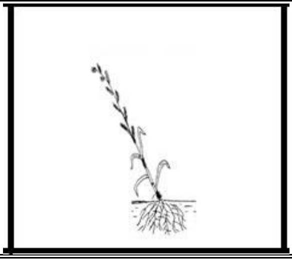 | 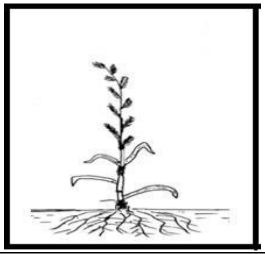 | |
| Your choice | | | | |

Card No. 7

| Attributes | Option A | Option B | Option C | Your current seed |
|---|---|---|---|---|
| Terminal heat during grain filling and milking stage($^0$C) | 30% yield loss<br>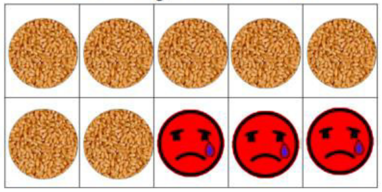 | 10% yield loss<br>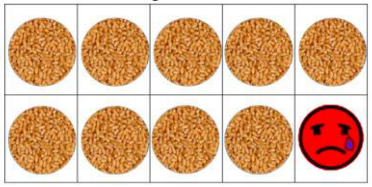 | 20% yield loss<br>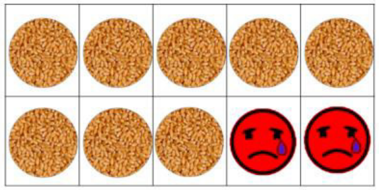 | |
| Potential yield (t/ha) | 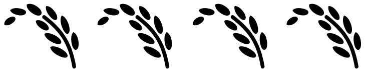 | 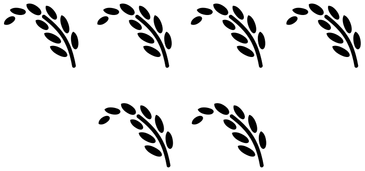 | 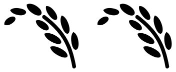 | |
| Chapati quality | 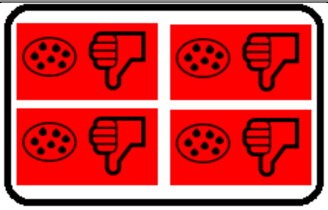 | 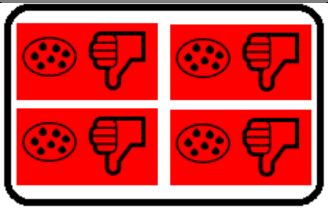 | 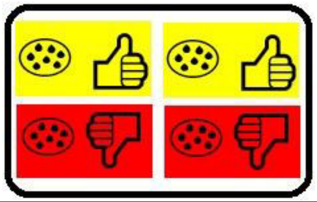 | |
| Seed price (Rs/kg) | 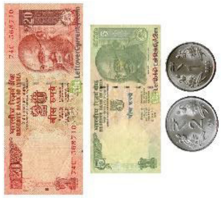<br>Rs. 28/- | 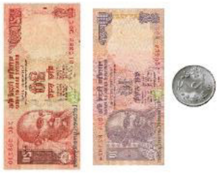<br>Rs. 32/- | 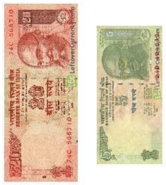<br>Rs. 25/- | |
| Lodging | 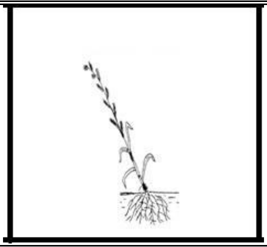 | 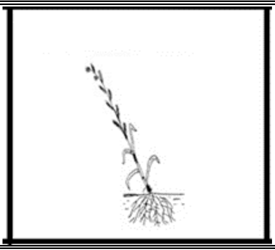 | 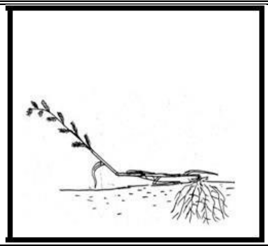 | |
| Your choice | | | | |

Card No. 8

| Attributes | Option A | Option B | Option C | Your current seed |
|---|---|---|---|---|
| Terminal heat during grain filling and milking stage($^{0}$C) | 20% yield loss 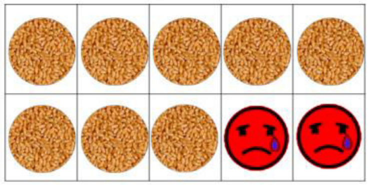 | 20% yield loss 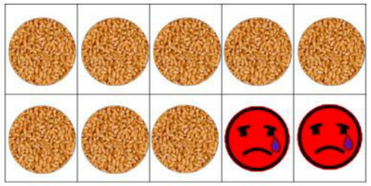 | 10% yield loss 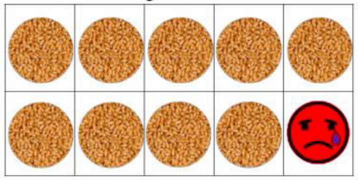 | |
| Potential yield (t/ha) | 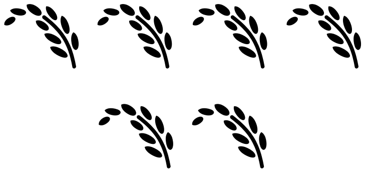 | 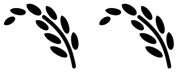 | 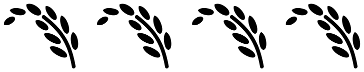 | |
| Chapati quality | 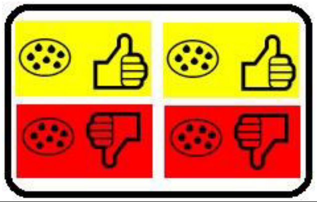 | 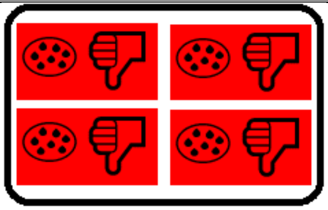 | 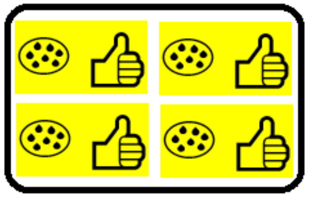 | |
| Seed price (Rs/kg) | 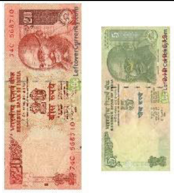 Rs. 25/- | 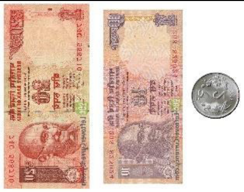 Rs. 32/- | 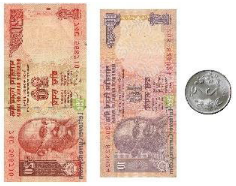 Rs. 32/- | |
| Lodging | 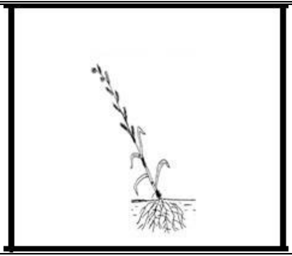 | 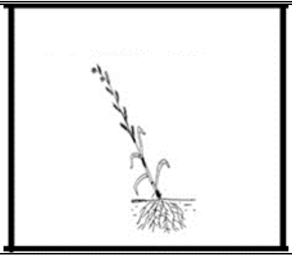 | 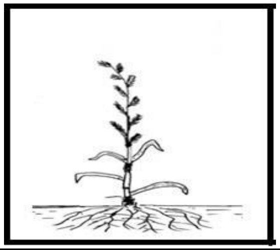 | |
| Your choice | | | | |

## Card No. 9

| Attributes | Option A | Option B | Option C | Your current seed |
|---|---|---|---|---|
| Terminal heat during grain filling and milking stage($^0$C) | 10% yield loss 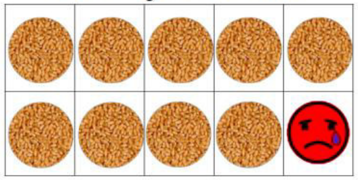 | 30% yield loss 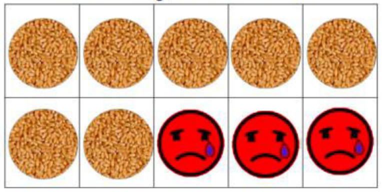 | 20% yield loss 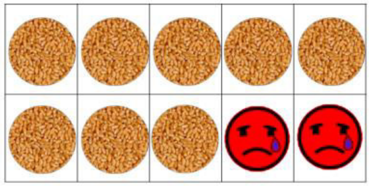 | |
| Potential yield (t/ha) | 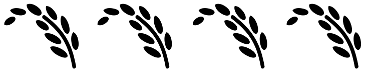 | 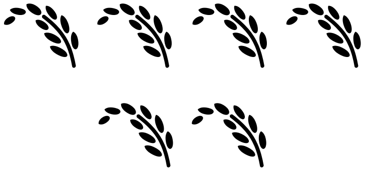 | 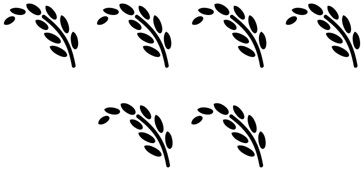 | |
| Chapati quality | 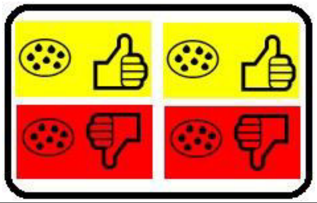 | 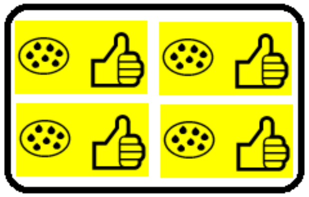 | 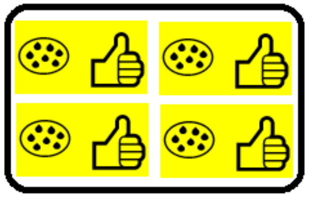 | |
| Seed price (Rs/kg) | 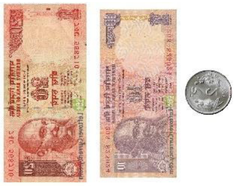 Rs. 32/- | 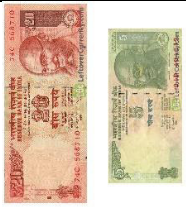 Rs. 25/- | 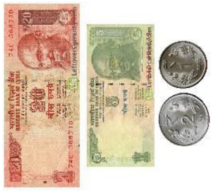 Rs. 28/- | |
| Lodging | 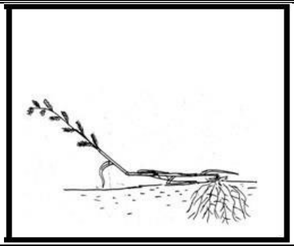 | 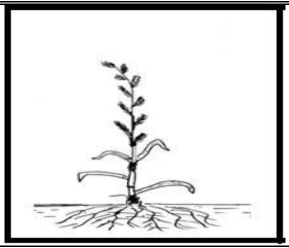 | 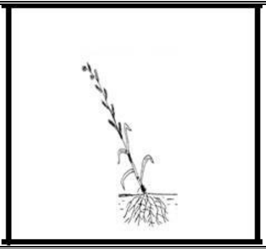 | |
| Your choice | | | | |
