# Supplementary material for: Gender, caste, and heterogeneous farmer preferences for wheat varietal traits in rural India
Source: PLoS One. 2022 Aug 11;17(8):e0272126. doi: 10.1371/journal.pone.0272126 (PMC9371340; doi:10.1371/journal.pone.0272126)
Supplement: Attachment — Submitted filename: Review report.pdf [file pone.0272126.s006.pdf]

Review report

Gender, Caste, and Farmer Preference for Wheat Varietal Traits in Rural India

1.1 Summary

The paper sets out to assess the preferences of central Indian farmers towards wheat varieties with a special attention to differences among gender and castes. Following an initial ranking exercise, a choice experiment involving five traits (heat tolerance, chapati quality, lodging, seed price and yield) is conducted; results are analysed through a mixed logit model correcting for attribute non-attention).

The paper makes some valuable contributions to studying farmers' preferences for varietal traits. Although the main analysis seems sounds, the presentation is not optimal leading to confusion. There are also some claims in the discussion that seem either unsupported by the data or vague/disconnected. I made several suggestions to clarify and sharpen the discussion but none of these are insurmountable. I highlighted the major comments in yellow.

1.2 References

- Reference 11 does not talk about farmer preference nor adoption but about the feasibility of increasing yield and quality at the same time
- P. 3 line 24: see also: *Paris, T. R., Singh, A., Cueno, A. D., & Singh, V. N. (2008). Assessing the impact of participatory research in rice breeding on women farmers: a case study in eastern Uttar Pradesh, India. Experimental Agriculture, 44(1), 97.*

1.3 Method

- P 22 l 6: you call the dataset a panel but as I understand it, the choice experiment is conducted in 2019 while some other data was already collected in 2018. I would, therefore, not call it a panel and also be more explicit about what is measured in 2018 (that is used in your study)
- You mention a preliminary survey: did this consist of only the ranking exercise or also other interventions? I think the words "preliminary survey" could be used in line 35 p 6 to contrast the words "main survey"
- I am confused to how the expert input (l 10 p 7) fits in the design of the choice experiment. Were the experts consulted to come up with

the list of 12 traits? If so, the text in lines 9-12 (up to " wheat varieties") on p 7 could be moved up before " The fieldwork of 2019 started with an". Or were the ranking and expert advice combined to come up with the final shortlist of 5 traits?

- L 34-38 p 7: I would start this paragraph with "When presented with non-familiar crop choices, lack of information about the costs and benefits affected the respondents' choices, resulting in high attribute non-attention (ANA)." and then be a bit more explicit about what ANA is and implies (I don't think it is common knowledge)
- The following sentence is unclear to me (l20-21 p 9): "Following Scarpa et al. [67], marginal WTP space is estimated for equations (3) and (4)." This paper discusses how to handle ANA while your study omits those observations with high or problematic ANA levels. How exactly does this study affect the way you calculated Tables 5 and 6?
- Regarding ranking analysis: a reference could be useful as this doesn't seem a standard technique, also it might be worth spelling out that for this metric a lower values reflects higher importance

1.4 Results

- Table 2:
  - the # symbol does not appear in the table footnotes.
  - Please also indicate what the numbers between brackets are (I assume sd).
  - Also rephrase the last sentence, perhaps into something like this: "*** and ** denote significance at the 5 and 10 per cent level compared to the Other castes group (non-marginalized), respectively"—Normally the more significant level (here: 5%) received the most stars, while you reverse this convention (assuming the software followed the normal convention, this seemed like a mistake but please check).
  - The sentence " If women were not responsible for decision-making, they could only contribute marginally to the decision-making process" (l 15-17 p 10) seems a bit empty. Wouldn't it be simpler to say: "Women being excluded from the decision-making process might explain why only 57% of women could name a major wheat variety ..."
- Table 3:
  - Why is the sample here not equal to 818?
  - I would drop " Decision-making on" from the table title, it is confusing as not every variable measured related to decision-making
  - " Decision-making by self": I'm unsure what this means exactly. Do you mean the only decision maker?

    - The category " If not self-decision-maker, involvement in decision making (scale 0-2)" is also confusing as the number of observations is no longer 740 and the mean of a categorical variable is not meaningful. I suggest to split out the different categories (0-2) and report the frequency of each. Table 1 from *Global survey of rice breeders to investigate characteristics and willingness to adopt alternative breeding methods* by Bert Lenaerts, Bertrand C. Y. Collard and Matty Demont is a good example of how to present binary and categorical data in one table
- Table 4
    - What does " Conditional on Importance==1" mean? Were traits only ranked for traits deemed important?
    - The significance stars are not correct
    - The "Importance [Dummy]" represents the frequency of this trait being stated important by farmers? If so, frequency might be clearer than "importance"
- Figure 2
    - I am not sure what this figure adds to the paper as almost all differences are not significant and the WTP values could be added to Table 5. I would delete this figure and just extend table 5 (see further)
- Order: I would change the order of figures and tables presented
    - Keep Appendix 1, Table 1, Figure 1 and tables 2-4
    - I would first introduce appendix 2 and then table 6: table 6 relies on interaction terms while table 5 actually runs regressions for subsets (male-female, castes), which is a more detailed approach (a subset approach is equal to taking the interaction with *every* variable included) so it should not come first. Then the ANA problem can be discussed too before moving on to table 5.
    - The inconsistency between tables 5 and 6 is also confusing, they could be put in the same layout. One suggestion to present this in a more comprehensible way could be to combine the mean and sd parameters in the upper part and the WTP parameters in the part below. A good example of this is Table 13 from *Willingness-to-pay estimation with mixed logit models: some new evidence* by Mauricio Sillano and Juan de Dios Ortu¨zar. If the table gets too long, the sd function might be dropped (or put in separate tables into on online appendix) since you don't discuss this in the main paper. This way we get the WTP for every model, which now is missing. That would again make Figure 2 redundant. Adding the units (per acre for

example, rs for WTP) is also useful as you refer to WTP per acre a few times.

- L 4-8 p 12: this paragraph confuses me and I'm not sure what it adds. It touches upon various concept likes agro-climate, strategy and selective breeding by farmers that are not essential to the choice experiment. It might improve the scope to simply delete this paragraph
- " Women considered a smaller set of attributes than men as important while making the varietal selection.": is this concluded from the lower frequencies? In that case, best to be specific and add that
- L 19-20 p 12: " However, men were found to be more likely to adopt varieties tolerant to heat stress and lodging than women" Here you could refer to the significant differences in ranking between men and women which reinforced this observation from table 5. This can be important to counter the argument that women have lower WTP because they have less agency over financial matters (and similar for the poorer castes) since the ranking is budget-independent.
- L 32-36 p 12: this is very vague, what does "preventing the expression" mean? That women have a lower WTP on average? Even if that is true, I don't think this justifies saying women cannot express their preferences. Also: why is the coefficient from Table 5 used to link the averages from Fig 2b? Line 34 refers to Fig 2b, however Table 6 shows parameters that are surprisingly similar in size between men and women (so contradicting this). I am also not sure that sensitivity to price (elasticity) is necessarily linked to WTP. I image that men might have greater decision-making power in financial matters (which you in fact show), which can explain both their (seemingly) higher WTP on average and their lower sensitivity to the price of seeds. Overall, I feel this paragraph muddies the results and should be left out or expanded upon to reflect the issues raised.

1.5 Discussion

- L 30 p 13: why is there a contrast ("despite") between having a large acreage and low productivity? I think the sentence would make more sense if the words " in terms of acreage in" are dropped
- L 35-40: this seems a repetition of the introduction, why repeat the aims and the fact that the topic is ill-studied?
- L 4-7 p 14: I don't see why this is added here? This more akin to food science and it is not clear how this information is used in the choice experiment.
- L 8-9 (begin): how was this calculated?
- L 15: source missing

- L 17: this seems an explanation for the contradiction raised earlier: only a quarter of varieties is premium in terms of grain quality, explaining why the region is still poor. I think it makes more sense to move this fact up to line 34 on the previous page
- L 24: higher than what? the market price at the private market?
- L25-27: how do you know this?
- L 2 p15: " portfolio of varieties preferred by them": do you mean the set of varieties actually used by the farmers?
- L 10: "realistic": do you mean that the set of varieties used reflects the ranking and WTP for different traits?
- L 19-20: this again needlessly restates the aim
- L 23-25: " it is perhaps surprising that men had a greater preference for new varieties that ensured high consumption utility. This was also reflected in the choice experiment estimates.": Does this refer to chapatti quality in Fig 2b? Because in my reading that is totally contradictory to the equal coefficients in table 5. I am not convinced that the paper provided evidence that men are more oriented towards marketed surplus. What you show is that men and women both value first chapati quality, then yield and only then risk-reducing factors. For the first two, there is no difference in both the ranking and table 5, only the risk factors differ between men and women. This follows from the data (you could speculate on the why part). The contradicting evidence in Figure 2b that men value chapatti quality more is interesting but without analysing this in more detail, I would not present this as a key finding. You can perhaps do a pairwise correlation analysis to see if underlying effect might explain this. But in the end, I think Table 5 is the more credible evidence to present.
- L 34: "low": add " lower than the other castes". I wonder whether this should be added given it is descriptive and insignificant, while for Table 5, the effects are sometimes higher than for other castes, so I am not sure the claim about " inability to pay in monetary terms " is justified. The ASC is definitely higher than for other castes so it seems there is higher WTP other varieties, which can be explained by the older varietal age (as you explain)
- L 3-6 p 16: this is just repeating what is said above
- L 28: " to farmers' less explored but important preferences": such as?

1.6 Conclusion

- Perhaps the implication of farmers' preferring chapatti quality above risk-reducing traits regarding climate change could be mentioned

1.7 Wording

- L 30 p3: "inter alia": "among other things" is a simpler alternative

- L 28-30 p 4: this seems a bit strongly formulated. I do agree that research on farmers' preference in wheat have focused on Ethiopia but saying that "we know hardly anything" seems exaggerated. A quick Google Scholar search already yielded two papers. I am not saying these should be added but the wording needs to be toned down:
    - Wheat Farmers' Preferences for Wheat Traits in Punjab, Pakistan A Choice Experiment Approach Hina Nazlia, Ekin Birolb, Dorene Asare-Marfob, and Asjad Tariqa
    - Farmers' Preferences of Improved Wheat Varieties in Wheat Subsector Kailali Nepal Prashant Bhatt1*, Pratibha Bist1, Laxmi Narayan Ojha
- L 7 p5: " on a par": equal with?
- " While caste-based social segregation is a unique feature of South Asian communities, its influence on millions of livelihoods and daily life is too strong to ignore": why is its uniqueness a reason to ignore it?
- P.5 l. 31: "often been examined": add "among different castes"
- L 24 p 11: at the end, add "with medium chapati quality"
- L 29 p 13: this is vague, what is fascinating? Also, you seem to solve the contradiction yourself (see l 17 p 14)
- L 9 p 14: the ";" at the end could be replaced by "as"
- L2 p 16: " is valuable for its uniqueness and" can be removed
