# Supplementary material for: Gender, caste, and heterogeneous farmer preferences for wheat varietal traits in rural India
Source: PLoS One. 2022 Aug 11;17(8):e0272126. doi: 10.1371/journal.pone.0272126 (PMC9371340; doi:10.1371/journal.pone.0272126)
Supplement: Attachment — Submitted filename: Reviewer_Report_PONE-D-21-06519.pdf [file pone.0272126.s007.pdf]

Reviewer’s report

Manuscript Number: PONE-D-21-06519

Title: Gender, Caste, and Farmer Preference for Wheat Varietal Traits in Rural India

Major comments

This paper examined farmers’ preferences for wheat varietal traits in Central India. The paper analyzed the preference heterogeneity across gender and caste. Authors used data collected from among 120 individuals from initial ranking, followed by a sex-disaggregated survey with a choice experiment among 420 farm-households in 2019. Authors concluded that developing and disseminating improved varieties with better grain quality and targeting women and marginalized social groups in varietal dissemination programs could enhance varietal turnover and wheat productivity in Central India. Overall, the research topic being addressed is interesting.

My major comments are as follows:

1. Authors developed an initial ranking of varietal traits followed by a household survey. However, there is no link between the attributes included in the ranking and the ones included in the choice experiment in the household survey. The link of the two pieces of survey is required to be mentioned in the manuscript.
2. The empirical methodology lacks explanation and confusion with trying to link the survey with panel data and the GENNOVATE project. Authors need to explain what the justification of the selection of three districts (Jabalpur, Damoh and Mandla) is. Similarly, how did the authors select 120 respondents for the ranking. It would have been good to conduct the ranking with the whole sample to avoid selection bias.
3. Authors aimed to analyse heterogeneity among gender and caste. Authors analyzed separately the gender and caste heterogeneities. However, this way will not allow to analyse gender heterogeneity within caste.
4. In the abstract, many results are not related findings of the manuscript. For instance, the result “Most women were not actively involved in varietal selection” (L16, p2) is not in the manuscript. Similarly, the result “marginalized caste and women farmers are open to experimentation with new varieties” (L16, p2) is not in the manuscript. In addition, the recommendation “targeting women and marginalized social groups in varietal dissemination programs could enhance varietal turnover and wheat productivity in Central India” (L22-24, p2) is not link to the result in the manuscript.
5. The data were collected in three districts in Central part of India where wheat is mainly produced for own consumption (l22, p14). Authors also recognize in the conclusion the particularity of the survey area. This limits the external validity of the findings in the manuscript. The findings may be less interested to international readers.

Minor comments:

1. Introduction: this study was conducted in a particular area with high poverty rate and the production of wheat is mainly for own consumption. This context is important to set the scene in the introduction.
2. Introduction: in line 24, add Khatri-Chhetri et al. (2017) after Arora et al. (2019).
3. Study area: The sentence “Gender inequality ………” (L18-20, p6) can be removed.
4. Sampling and data collection: Why authors decide to choose three villages where GENNOVATE project is working? The intervention of GENNOVATE may have biased the perception of participants compare to other villages where GENNOVATE is not intervened.
5. Results should not be presented in the methodology (L35-39, p6).
6. L38 (p6), explain how 12 attributes were selected?
7. Choice experiment design: Authors mentioned that the discussion with local expert was carried out to identify the key attributes for the choice experiment. What is the role of the ranking done with 120 farmers?
8. L16 and 17 (p7), the words familiar and unfamiliar should be part of the results.
9. L25 (p7), present in appendix the nine choice sets selected.
10. L26-27 (p7), please justify why you choose three alternatives scenario for each farmer. How the choice of set to a farmer is done?
11. Econometric estimation of the WTP: add to this section the expression for the calculation of the WTP. It is missing.
12. Equation 1 only considers the gender disaggregation. What is about the caste disaggregation?
13. Equation 3, what is the rational of the selection of the variables in this equation? Why not consider other attributes use in the ranking?
14. Equation 4: Why the caste variables SC and ST are separated in equation 4 while they are combined in other part of the manuscript (for instance in Table 5). How food security variable is calculated?
15. L15-21 (p9): the reason of using WTP space approach is not clear. In addition, the supporting references are all old with more than 10 years. This implies that WTP space approach is not new as such. Why the authors want to use this method?
16. Results: Because there is a section on discussion, all discussions need to be removed from the results section (for instance L33-34 (p9), L3-4 (p10))
17. L23-31 (p12) should be in the methodology.
18. L32-33 (p12), there is no seed cost in Fig 2b.
19. L36 (p12), please explain “the relatively flat demand curve…” Where is the demand curve? Similar explanation is needed for the sentence on L29-31 (p15).
20. Discussion and implications: the link between poverty and cultivation of wheat varieties known for premium quality is not obvious. The introductory part of the discussion section (L29-35, p13) should be moved to the introduction section.
21. Table 2: Add the disaggregation by gender in this table. Because the adoption rates varied by varieties, the weighted average should be used to calculate the average varietal age of

release? How is the age of unknown varieties is being considered in the estimation of the average varietal age of release? The value (17.51) stands for what?

22. Table 3: should be presented based on caste disaggregation
23. Table 4: what test is performed for comparison?
24. Table 5: The results of the mixed logit model need to be analysed in detail. For instance, what is the meaning of the Standard deviation function in Table 5.
25. Table 6: The variable female was not in equation 4. Specify the meaning of WTP in Rs. Give the source of measing food security with scale from 0-8?
26. Appendix I: Add the status quo
27. Appendix III: can be removed.
