# Supplementary material for: Gender, caste, and heterogeneous farmer preferences for wheat varietal traits in rural India
Source: PLoS One. 2022 Aug 11;17(8):e0272126. doi: 10.1371/journal.pone.0272126 (PMC9371340; doi:10.1371/journal.pone.0272126)
Supplement: Attachment — Submitted filename: Response to Reviewer 2.pdf [file pone.0272126.s008.pdf]

Response to the Reviewers on Manuscript Titled "Gender, Caste, and Farmer Preference for Wheat Varietal Traits in Rural India" (PONE-D-21-06519)

We, the authors, are grateful for the comments made by the two reviewers, which have helped us further improve the quality of our manuscript. Below we describe how we have addressed each comment. For convenience, we cite the original comments, before responding.

Response to Reviewer 2

Comment 1: *This paper examined farmers' preferences for wheat varietal traits in Central India. The paper analyzed the preference heterogeneity across gender and caste. Authors used data collected from among 120 individuals from initial ranking, followed by a sex-disaggregated survey with a choice experiment among 420 farm-households in 2019. Authors concluded that developing and disseminating improved varieties with better grain quality and targeting women and marginalized social groups in varietal dissemination programs could enhance varietal turnover and wheat productivity in Central India. Overall, the research topic being addressed is interesting.*

Response: The summary of the study provided by Reviewer 2 is concise, and we would thank the reviewer for his time and valuable suggestions and positive evaluation. Below we describe how we have addressed each comment. For convenience, we cite the original comments, before responding.

Comment 2: *Authors developed an initial ranking of varietal traits followed by a household survey. However, there is no link between the attributes included in the ranking and the ones included in the choice experiment in the household survey. The link of the two pieces of survey is required to be mentioned in the manuscript.*

Response: We agree with the reviewer that in the previous draft we didn't specify how the ranking exercise was linked to the choice modelling. We found that it cannot be done in the methodology section as the results of the ranking exercise have to be elaborated. Now we have added a paragraph in the results section as follows (Page 12, Lines 9-25):

"According to the ranking exercise, the two most important characteristics of wheat varieties were related to quality – good for *chapatis* (taste, puffiness, etc.) and color of grains at maturity (light-colored, white or yellow). About 94% of respondents found these traits important in varietal selection. Big/bold grain, a composite trait of quality and quantity, was important for 86%. Length and spread of awns – another composite trait of quality and quantity (awn spread indicates boldness of grains) and risk aversion (grains with long awns have high pest resistance according to farmers) – were also ranked high. We included the most prominent quality variable (*chapati* quality) in the choice experiment, being a most prominent trait in decision-making. After quality, farmers ranked traits indicative of higher yield (height of the plant, more tillers per plant) above

traits showing risk reduction (tolerance to drought, lodging, and diseases). During both the ranking exercise and the piloting of choice experiments, many farmers opined that 'grain yield in general' was more important in the variety selection than the individual yield components like the number of tillers. Based on this information, a quantity trait was included in the choice experiment: 'grain yield per land unit'. We included two risk-ameliorating factors from the ranking exercise – lodging tolerance (a familiar, high ranked trait) and terminal heat tolerance (an unfamiliar, low ranked trait). Because heat tolerance is expected to play an increasingly important role in wheat breeding, we provided an information priming on this trait before presenting farmers with the alternatives to choose."

Comment 3: *The empirical methodology lacks explanation and confusion with trying to link the survey with panel data and the GENNOVATE project. Authors need to explain what the justification of the selection of three districts (Jabalpur, Damoh and Mandla) is. Similarly, how did the authors select 120 respondents for the ranking. It would have been good to conduct the ranking with the whole sample to avoid selection bias.*

Response: We agree that we have to provide more information on the selection of districts. Now we have revised the text as follows.

(a) *Selection of study districts*. "was included only in 2019-round, we excluded the first-round dataset from the main analysis. Data collection was confined to the rural villages of Jabalpur, Mandla, and Damoh districts, where wheat is widely cultivated. Most of the existing socioeconomic analysis of wheat cultivation in India is either from the western states of the IGP, such as Punjab and Haryana, or from the eastern states, such as Bihar. The study districts were selected purposively, recognizing the knowledge gap on wheat cultivation on the subsistence farming systems of Central India. The selected districts present an interesting spectrum of social diversity, where the aboriginal (tribal) households and non-tribal households live in close geographical proximity [54]. The social norms that vary across the caste groups could influence the gender relations in farm production." (Page 6 Lines 27-35).

(b) *Overlap with GENNOVATE project*. "In addition to these fifteen randomly selected villages, three villages were included where qualitative gender studies were completed in 2014 and 2015, as part of a project, GENNOVATE (https://gennovate.org/), which was a large-scale qualitative comparative research initiative to analyze how the nexus between gender norms, women's agency and innovation in agriculture influenced different innovation processes across diverse contexts [57]. An overlap of the study villages was expected to generate useful insights on the locally prevailing social norms from the existing GENNOVATE case-studies. From all the villages, wheat farming households were selected randomly, after completing a census of farm households. In total, 28 farm households were included from each of the nine large villages (336 households) and 14 households from each of the six small villages (84 households). None of the GENNOVATE focus group discussants fell in the random sample of households (2018)." (Page 7 Lines 2-12).

(c) *Selection of 120 farmers for ranking exercise.* "The fieldwork of 2019 started with a preliminary ranking exercise of varietal traits among a sub-sample of 120 farmers, randomly selected from the 2018 respondent list." (Page 6, Lines 13-14).

Comment 4: *Authors aimed to analyse heterogeneity among gender and caste. Authors analyzed separately the gender and caste heterogeneities. However, this way will not allow to analyse gender heterogeneity within caste.*

Response: In one of the earliest drafts of the paper, we had gender*caste intersectionality, which was eventually dropped and did not appear in the submitted version due to statistical insignificance. However, based on the reviewer's comment, we realize now that the story is pertinent, and hence included interaction of gender dummy (=1 if female) with caste (SCST dummy) and economic status (i.e., food insecurity). We found that the Gender*SCST interaction is statistically insignificant, while Gender*Food_insecurity interaction affects farmer preferences strongly. Kindly refer to Table 6 for the revised estimates.

Comment 5: *In the abstract, many results are not related findings of the manuscript. For instance, the result "Most women were not actively involved in varietal selection" (L16, p2) is not in the manuscript. Similarly, the result "marginalized caste and women farmers are open to experimentation with new varieties" (L16, p2) is not in the manuscript. In addition, the recommendation "targeting women and marginalized social groups in varietal dissemination programs could enhance varietal turnover and wheat productivity in Central India" (L22-24, p2) is not link to the result in the manuscript.*

Response: We agree that the findings stated in the abstract were not explicit in the main text. We added a table in Appendix 2 to support Table 3 to show that "Most women farmers were not actively involved in making decisions related to wheat cultivation, including varietal selection" (Page 2 Lines 16-17).

Regarding the second point, we found that women and socially marginalized caste households have a higher willingness to adopt new varieties (Table 6), which leads to a natural conclusion that, "From the observed patterns, one could deduct that developing and disseminating improved varieties with better grain quality and targeting women and marginalized social groups in varietal dissemination programs could enhance farmer adoption of new, improved germplasm and wheat productivity in Central India" (Page 2, Lines 21-24). We have revised the results and discussion in several parts to state these points clearly (e.g., Section 5.3).

Comment 6: *The data were collected in three districts in Central part of India where wheat is mainly produced for own consumption (l22, p14). Authors also recognize in the conclusion the particularity of the survey area. This limits the external validity of the findings in the manuscript. The findings may be less interested to international readers.*

Response: Thank you for pointing out this special feature of our study. It is now added in the description of the study area: "The study districts were selected purposively, recognizing the knowledge gap on wheat cultivation on the subsistence farming systems of Central India. The selected districts present an interesting spectrum of social diversity, where the aboriginal (tribal) households and non-tribal households live in close geographical proximity [54]. (Line 31-34, Page 6)".

In the conclusion section, we have explicitly stated the limitation of our study. "Finally, there are certain limitations to generalizing these findings to apply to a wider farmer population. The sample is limited to three districts of Central Madhya Pradesh, where the wheat production scenario is unique for the predominance of consumption utility in varietal selection. Farmers in other states such as Punjab and Haryana, India's breadbasket with well-integrated markets, have a relatively rapid varietal replacement and possibly different preferences for wheat varietal traits. Therefore, caution may be exerted in generalizing the findings. Furthermore, there could be variations in the nature and implications of gender- and caste-based preference heterogeneities across Indian states. While recommending for a more participatory, farmer-oriented approach in developing the crop breeding programs, we also recognize the need for more socioeconomic research studies to be conducted across the wheat systems India." (Page 18, Lines 6-15).

Comment 7: *Introduction: this study was conducted in a particular area with high poverty rate and the production of wheat is mainly for own consumption. This context is important to set the scene in the introduction.*

Response: As indicated as response to Comment #6, we have indicated the uniqueness of the study area with respect to subsistence consumption. The revised text includes the points noted by the reviewer in the introduction section. "We will see in the forthcoming sections that a large share of the wheat production by sample households – especially among the income-poor – goes for home consumption, making consumption utility an integral part of the decision-making over varieties. The popularity of some of the old wheat varieties (e.g., Lok-1, released in 1982) in the region has been attributed to their suitability for making tasty *chapatis* (and *chapati* variants such as *roti, paratha, phulka,* etc.) [26,27]. However, no study has established the connection between farmers' preference for consumption traits and the prevalence of old varieties, particularly among the income poor and food insecure, the socially marginalized, and women farmers." (Page 3 Lines 40 to Page 4 Lines 1-6).

Comment 8: *Introduction: in line 24, add Khatri-Chhetri et al. (2017) after Arora et al. (2019).*
Response: We have followed this sugession while revising the text. (Page 3, Line 24).

Comment 9: *Study area: The sentence "Gender inequality ………" (L18-20, p6) can be removed.*
Response: We have followed this sugession while revising the text.. (Page 3, Line 22).

Comment 10: *Sampling and data collection: Why authors decide to choose three villages where GENNOVATE project is working? The intervention of GENNOVATE may have biased the perception of participants compared to other villages where GENNOVATE is not intervened.*
Response: We understand the reviewer's concern, but we would politely disagree here. There are two reasons. One, the GENNOVATE case studies were conducted 4-5 years

before the quantitative survey. Also, GENNOVATE did not do any interventions; the researchers did some in-depth interviews and FGDs only. Secondly, we found that none of the GENNOVATE focus group discussants fell in the random sample of households. We have added details of GENNOVATE project in Section 3.2 (Page 7).

Comment 11: *Results should not be presented in the methodology (L35-39, p6). 6. L38 (p6), explain how 12 attributes were selected?*

Response: These findings are now shifted to results. Please see Page 12 Lines 3-5.

Comment 12: *Choice experiment design: Authors mentioned that the discussion with local expert was carried out to identify the key attributes for the choice experiment. What is the role of the ranking done with 120 farmers?*

Response: We have elaborated the role of local experts in Page 7 Lines 28-32 in the revised manuscript as follows. "One of the necessary steps in designing a choice experiment is to identify the relevant attributes (varietal traits) for each alternative and levels of each attribute considered for the experiment [60,61]. Discussions with local experts were carried out to understand the possible reasons for farmer preference for certain traits in the ranking exercise, and to fix realistic levels of each of the selected attributes. Using the insights, five traits were included in the choice experiment."

Comment 13: *L16 and 17 (p7), the words familiar and unfamiliar should be part of the results.*

Response: We agree. These words familiar/unfamiliar and high/low-ranking terms were removed from the methodology (Page 7). The rationale for including these traits is now shown in the results section (Page 12 Lines 21-23). "We included two risk- ameliorating factors from the ranking exercise – lodging tolerance (a familiar, high ranked trait) and terminal heat tolerance (an unfamiliar, low ranked trait)."

Comment 14: *L25 (p7), present in appendix the nine choice sets selected.*

Response: An example choice card is provided in Appendix I and the complete set in the Online Supplementary Materials S1 and indicated about it in Page 8 Lines 19-20.

Comment 15: *L26-27 (p7), please justify why you choose three alternatives scenario for each farmer. How the choice of set to a farmer is done?*

Response: We have addressed this concern in the text as follows. "Each choice set offered respondents an option to choose from four alternatives (three product profiles and the status quo), resulting in a multidimensional choice-making scenario where respondents considered all attributes simultaneously. In the piloting stage, we found that increasing the alternatives per choice set above three (plus status quo option) significantly added interview time. Providing two alternatives and the status quo was also

considered. However, we followed the suggestion from the literature that providing three alternatives could yield more robust models than two [62]. When two alternatives are provided, serial non-participation – "choosing an alternative consistently without regard for changes in the attributes" (ibid; p. 1140) – was found occurring." (Page 8 Lines 7-14).

[62] Rolfe J, Bennett J. The impact of offering two versus three alternatives in choice modelling experiments. Ecological Economics. 2009; 68:1140–8. doi: 10.1016/j.ecolecon.2008.08.007.

Comment 16: *Econometric estimation of the WTP: add to this section the expression for the calculation of the WTP. It is missing.*

Response: Thank you for this suggestion, following which we have included the econometric estimation of the WTP (Eq. 5 and 6) in Page 10.

Comment 17: *Equation 1 only considers the gender disaggregation. What is about the caste disaggregation?*

Response: Again, thank you for noting this mistake. The section is modified as follows:

"Hypothesizing that the utility function is systematically different for men and women (or for marginalized and non-marginalized castes), we developed the following econometric specification:

$$U_{ijt} = V(Z_{ijt}, P_{ijt}, S_i) + \varepsilon(Z_{ijt}, P_{ijt}, S_i), \qquad j \in \boldsymbol{C} \text{ and } U_{ijt} = \begin{cases} U_{1jt} \\ U_{2jt} \end{cases} \qquad \text{(Eq. 1)}$$

For any (male or female / marginalized caste or otherwise) farmer i, a given level of utility is associated with the choice of an alternative wheat variety j in choice set series t. Alternative *j* is chosen over some other alternative k in a given choice set $\boldsymbol{C}$, if and only if $U_{ij} > U_{ik}$ for all j, $k \in \boldsymbol{C}$. The utility U_{1jt} and U_{2jt} can either represent the male/female or marginalized/non-marginalized caste category duos." (Page 9 Lines 1-8).

Comment 18: *Equation 3, what is the rational of the selection of the variables in this equation? Why not consider other attributes use in the ranking?*

Response: We have to limit the number of attributes to be included in the choice experiment to obtain robust WTP estimates. The choice of attributes was justified in the result section as follows:

"According to the ranking exercise, the two most important characteristics of wheat varieties were related to quality – good for *chapatis* (taste, puffiness, etc.) and color of grains at maturity (light-colored, white or yellow). About 94% of respondents found these traits important in varietal selection. Big/bold grain, a composite trait of quality and quantity, was important for 86%. Length and spread of awns – another composite trait of quality and quantity (awn spread indicates boldness of grains) and risk aversion (grains with long awns have high pest resistance according to farmers) – were also ranked high. We included the most prominent quality variable (*chapati* quality) in the choice experiment, being a most prominent trait in decision-making. After quality, farmers

ranked traits indicative of higher yield (height of the plant, more tillers per plant) above traits showing risk reduction (tolerance to drought, lodging, and diseases). During both the ranking exercise and the piloting of choice experiments, many farmers opined that 'grain yield in general' was more important in the variety selection than the individual yield components like the number of tillers. Based on this information, a quantity trait was included in the choice experiment: 'grain yield per land unit'. We included two risk-ameliorating factors from the ranking exercise – lodging tolerance (a familiar, high ranked trait) and terminal heat tolerance (an unfamiliar, low ranked trait). Because heat tolerance is expected to play an increasingly important role in wheat breeding, we provided an information priming on this trait before presenting farmers with the alternatives to choose." (Page 12 Line 9-25).

Comment 19: *Equation 4: Why the caste variables SC and ST are separated in equation 4 while they are combined in other part of the manuscript (for instance in Table 5). How food security variable is calculated?*

Response: '*SC and ST are separated in equation 4*' – it was an inconsistency in the earlier draft, and we thank the reviewer for noting it. We have revised tables and text with SC and ST combined.

"Food insecurity was estimated with Household Food Insecurity Access Scale, which is commonly used to measure household food insecurity [66,67]". (Page 9 Lines 30-32).

Comment 20: *L15-21 (p9): the reason of using WTP space approach is not clear. In addition, the supporting references are all old with more than 10 years. This implies that WTP space approach is not new as such. Why the authors want to use this method?*

Response: Among the two main approaches of WTP estimation – preference space approach and WTP space approach – we followed the latter. We have now added the following explanation to justify the selection. "Deviating from the traditional approach of calculating the willingness to pay (WTP) for an attribute and employing the extension of parameter space to the WTP space approach suggested by Train and Weeks [71] and Sonnier et al. [72], we allowed random parameters (and, consequently, random WTP measures) in the estimation. This approach, as it accounts for the preference heterogeneity [71], is more suitable for the current study over the alternative (the preference space approach)." (Page 10 Lines 16-20).

Comment 21: *Results: Because there is a section on discussion, all discussions need to be removed from the results section (for instance L33-34 (p9), L3-4 (p10)) 17. L23-31 (p12) should be in the methodology.*

Response: Following reviewers suggestion, we have removed all discussions from the results section. For example –

> "We found that the average age of varieties cultivated by sample farmers was 30 years, which is extremely high (and hence undesirable) as compared to the average varietal age of wheat in India (10-12 years) and the age recommended to curtail the risks associated with wheat rust (<6 years) [23]. This low turnover makes the system highly vulnerable to biotic stresses like rust.". This section is now in Discussions (Page 15 Lines 23-27).

[*Old text*] “Limited access to formal and informal information networks for marginalized caste farmers, as shown by Krishna et al. [48] and Gupta et al. [68], could be contributing to the low adoption of new varieties.” – This part is now deleted.

The mixed logit models were estimated with interaction terms to compare the average WTP of women with that of men. “Here, the ASC is interacted with the female dummy variable. A positive ASC interaction with a household attribute would imply that if the respondent did not choose the status quo option in the choice experiment, he or she would derive a positive utility from adopting the new varieties and be willing to pay for them.” – This part is now in the methodology section (Page 10 Lines 11-15).

We have made more editing to remove the out-of the place sentences from the results section.

Comment 22: *L32-33 (p12), there is no seed cost in Fig 2b. 19. L36 (p12), please explain “the relatively flat demand curve…” Where is the demand curve? Similar explanation is needed for the sentence on L29-31 (p15).*

Response: We have revised the text and it appears as follows now. “Women also faced a steeper demand function as compared to men, as evidenced by the highly negative coefficient of seed price (-0.005 *vs*. men’s -0.001), showing that women’s WTP for new varieties drop drastically with an increase in the seed price.” (Page 12 Line 38 to Page 13 Line 2).

Comment 23: *Discussion and implications: the link between poverty and cultivation of wheat varieties known for premium quality is not obvious. The introductory part of the discussion section (L29-35, p13) should be moved to the introduction section.*

Response: We have revised the text as follows: “Madhya Pradesh has long remained in the second position with respect to wheat acreage in India [74]. As shown in Section 3.1, the wheat productivity in the state is lower than the national average, one of the reasons for which could be the suboptimal varietal turnover rate. We found that the average age of varieties cultivated by sample farmers was 30 years, which is extremely high (and hence undesirable) as compared to the average varietal age of wheat in India (10-12 years) and the age recommended to curtail the risks associated with wheat rust (<6 years) [23].” (Page 15 Lines 21-26). We have taken out the link of poverty and cultivation of wheat varieties from this part as per the reviewer’s suggestion.

Comment 24: *Table 2: Add the disaggregation by gender in this table. Because the adoption rates varied by varieties, the weighted average should be used to calculate the average varietal age of release? How is the age of unknown varieties is being considered in the estimation of the average varietal age of release? The value (17.51) stands for what?*

Response: Unfortunately, the wheat cultivation in India is done jointly by men and women and gender disaggregation is not possible. We have used the weighted averages. Doing the follow up field work, we could avoid the incidents of unknown varieties, and hence excluded from the table. Values in the parentheses shows standard deviation values. The procedure of taking weighted average and the details of figures in the parentheses are now shown in the table footnote.

Comment 25: *Table 3: should be presented based on caste disaggregation*

Response: Thank you for this suggestion. We have followed it in the revised draft.

Comment 26: *Table 4: what test is performed for comparison?*

Response: The inter-group equality of proportions and means is tested using large-sample statistics (*prtest* for importance dummy and *ttest* for ranks; Stata 16.0). This detail is shown as table footnote now.

Comment 27: *Table 5: The results of the mixed logit model need to be analyzed in detail. For instance, what is the meaning of the Standard deviation function in Table 5.*

Response: We have now added the following sentences. "The standard deviation parameters are statistically significant for three attributes – yield, high chapati quality, and seed price – indicating the existence of preference heterogeneity." (Page 12 Lines 32-35). To maintain the word limit, we couldn't further detail the meaning of standard deviation. Furthermore, the coefficients of the mixed logit model are not directly interpretable. Hence, we focused our discussion on the estimated WTP.

Comment 28: *Table 6: The variable female was not in equation 4. Specify the meaning of WTP in Rs. Give the source of measuring food security with scale from 0-8?*

Response: We have added the variable "gender" (and its interaction terms) in Eq. 4 now. We have added two new paragraphs on the estimation of the WTP values (Page 10 Lines 16-33).

Two references are provided as the source of measuring food insecurity.

- Coates J, Swindale A, Bilinsky P. Household Food Insecurity Access Scale (HFIAS) for Measurement of Food Access: Indicator Guide (v. 3). Washington DC: Food and Nutrition Technical Assistance Project, Academy for Educational Development; 2007.
- Knueppel D, Demment M, Kaiser L. Validation of the Household Food Insecurity Access Scale in rural Tanzania. Public Health Nutrition. 2010; 13:360–7. Epub 2009/08/26. doi: 10.1017/S1368980009991121 PMID: 19706211.

Comment 29: *Appendix I: Add the status quo*

Response: The example choice card is provided with status quo option in the Appendix.

Comment 30: *Appendix III: can be removed.*

Response: Following the reviewer suggestion, Appendix III is removed.

Finally, we would like to thank the reviewer the valuable suggestions and dedication to comment extensively.
