# Supplementary material for: Gender, caste, and heterogeneous farmer preferences for wheat varietal traits in rural India
Source: PLoS One. 2022 Aug 11;17(8):e0272126. doi: 10.1371/journal.pone.0272126 (PMC9371340; doi:10.1371/journal.pone.0272126)
Supplement: Attachment — Submitted filename: Response to Reviewer.docx [file pone.0272126.s009.docx]

**Response to Reviewer 1 on the manuscript titled "Gender, Caste, and Farmer Preference for Wheat Varietal Traits in Rural India" (PONE-D-21-06519R1)**

*Comment 1*: The authors clearly improved the manuscript and added additional information. However, some ambiguity remains and should be addressed before the paper can be considered for publication.

*Response*: We would like to sincerely thank the reviewer for the scrupulous review, which has helped us improve the manuscript's quality. Below we describe how we have addressed each comment. For convenience, we cite the original words before responding.

*Comment 2* (Section 3): I am still confused by the data sampling process. You say you rely on a previously conducted CIMMYT study, but a number of things are missing:

- Reference to this study (even if only to a working paper)
- What kind of information is used from this CIMMYT study? You explain a sampling strategy, but it is unclear whether you or the CIMMYT study has done this. You also seem to refer to households and the "2018 respondent list", but later on, you refer to "after completing a census of farm households": why is this census needed and when was it conducted?

*Response*: We agree with the reviewer that the description of the sampling strategy was confusing in the earlier draft. We have now referenced two studies that used 2018 survey data (Surendran-Padmaja et al., 2021 and Farnworth et al., 2022 – references numbers in the manuscript [58, 59]). We have also replaced the "CIMMYT researcher" with "the first author of the present study." In the revised draft, we first elaborated on the sampling procedure followed in the 2018 round (including the timing of the village census) and then explained the second round of the survey. Please refer to the first two paragraphs of Section 3.2 (Pages 6-7) for the changes made. Later, we explained that the first-round dataset was excluded from the main analysis because the focus was on farmer preference for wheat varieties (which is included only in the 2019-round). We hope that these changes help the reader better understand the sampling procedure.

*Comment 3* (Section 3): You also state that "we excluded the first-round dataset from the main analysis" but later on add that "We supplement Table 3 with data on gendered participation in wheat varietal selection, using the 2018 survey data". This seems contradictory.

*Response*: We agree with the reviewer – there were two contradicting statements. Because the choice experiment on farmer preference for wheat varieties was included only in the 2019-round, we excluded the 2018 dataset from the main analysis. In other words, both dependent and explanatory variables of the choice experiment were derived only from the 2019 dataset. However, 2018-round data were used for complementary analysis on gendered participation in wheat varietal selection. This analysis provided the necessary contextual reasoning for the heterogeneity in the trait preferences. We have included these details now in the revised version (Section 3.2; Page 7).

*Comment 4* (Section 3): The sentence "The social norms that vary across the caste groups could influence the gender relations in farm production." Is redundant.

*Response*: This sentence is now deleted from the text. Please refer to Page 6.

*Comment 5* (Section 3): Eq. 1, 2, 5 and 6 seems not strictly necessary: the most important is to show your final formula to be estimated (Eq. 3-4) and how the WTP is calculated (ratio of trait coefficient over seed price coefficient). The use of too many Greek symbols is lowering the readability. Also, p. 16 (manuscript numbering) lines 9-10 seems to contradict line 18: are the parameters assumed random or not?

*Response*: Following the reviewer's suggestion, we have deleted the equations (1), (2), (5), and (6), and modified the explanation accordingly.

[We assume that the reviewer refers to p.10 in this comment (as on page 16, the discussion is not about the randomness of parameters]. In lines 9-10 (of the previous draft), it was written that "We assumed that preference heterogeneity exists, and thus the attributes are specified randomly and normally distributed" and in line 18 that "we allowed random parameters (and, consequently, random WTP measures) in the estimation." These statements suggest that heterogeneity exists, and thus a random parameter is more appropriate. We do not think they contradict each other.

*Comment 6* (Section 4): "In our survey, the male respondents also did not acknowledge the role of women members": what does this mean and does this apply to every single male respondent in the entire sample?

*Response*: We have modified this section as follows (Page 11).

"… most male respondents did not acknowledge the role of women members of the household in the discussions on varietal selection. For instance, women respondents from 144 households indicated that they participated in the discussions related to varietal selection. In about 58% of these cases, the male respondents denied their involvement. Due to a lack of agency and male household heads' denial of their involvement in varietal selection, women farmers' varietal preferences might not be fully expressed in the adoption statistics..."

*Comment 7* (Section 4): Table 4: " inter-group equality": male vs female, SCST vs other castes?

*Response*: Thank you again for being careful, thorough, and attentive to details. We have changed the description as follows. "The inter-group (SCST vs. Other castes and Men vs. Women) equality of proportions and means is tested using large-sample statistics"….

*Comment 8* (Section 4): P 19 l 7-8: "There exists significant preference heterogeneity within the gender groups, as shown by the statistically significant standard deviation parameter for most of the traits.": this seems confusing for two reasons:

- I only see a difference in standard deviation for chapati quality, so the statement seems an overgeneralization
- "Preference heterogeneity within the gender groups" is also confusing; it might be more accurate to say something like: Female respondents expressed a significantly higher variability in preference for high Chapati quality than male respondents, and oppositely for medium Chapati quality.

*Response*:­ We agree that there was confusion, and thanks to the reviewer for pointing this out. There are two types of heterogeneities we wanted to highlight here – within and between male and female respondents. We have changed the description as follows. "The standard deviation function indicates that female respondents expressed a significantly greater variability in preference for medium *chapati* quality than male respondents, whereas the opposite holds for high *chapati* quality. Male respondents expressed a large variability in preference for potential yield and medium lodging traits. Non-marginalized caste farmers showed greater variability than SCST farmers in preference for medium lodging." (Page 12).

*Comment 9* (Section 4): P19 l 17-19: "The results show that the effect of excluding this minority, and therefore the effect of ANA, is highly pronounced in the preference estimates of all traits included in the choice experiment.": the term highly pronounced seems exaggerated: only high Chapati quality, high lodging tolerance and ASC is significantly affected.

*Response*:­ With due respect, we disagree with the reviewer's observation here. Even with a slight change in the coefficient of seed price, the WTP estimates change significantly. For example, farmers' WTP for high tolerance and medium tolerance increased by 48% and 60%, respectively. For clarity, we changed the sentence as follows. "The results show that the effect of excluding this minority, and therefore the effect of ANA, is highly pronounced in the WTP estimates of all traits included in the choice experiment." (Page 12).
